# Community Specialist Teams for Older People Consensus Development: A Real-Time Delphi Approach

**DOI:** 10.5334/ijic.8781

**Published:** 2025-10-21

**Authors:** Christine FitzGerald, Christina Hayes, Aoife Whiston, Jennifer Hardiman, Marian Mullaney, P. J. Harnett, Patrice Reilly, Brian Condon, Alison Holmes, Íde O’Shaughnessy, Collette Devlin, Louise Barry, Katie Robinson, Emer Ahern, Rose Galvin

**Affiliations:** 1 School of Allied Health, Ageing Research Centre, University of Limerick, Ireland; 2 Department of Psychology, University of Limerick, Ireland; 3 National Clinical Programme for Older People, Ireland; 4 Mowlam Healthcare, Limerick, Ireland; 5 National Clinical Advisor and Group Lead for Older Persons, Cork, Ireland; 6 Department of Nursing and Midwifery, University of Limerick, Ireland

**Keywords:** Community Specialist Teams for Older People, consensus development, Delphi method

## Abstract

**Background:**

This study aimed to develop consensus on the core elements of the Community Specialist Teams for Older People (CST OP) service model.

**Methods:**

This study utilised a modified Delphi approach. World Café Focus Groups (n = 97) were facilitated to develop and refine statements which were included in a Real-Time Delphi survey.

**Results:**

Four key themes were produced from the World Café Focus Groups data: Fundamentals of service design, Access and transitions of care, Team Functions and Quality improvement. Themes produced informed the CST OP Real-Time Delphi survey, which was completed by 77 participants, representing a response rate of 78%.

**Conclusion:**

The modified Delphi approach has resulted in the refinement and agreement of a set of core CST OP statements that outline the key components of the CST OP service model which will guide future implementation and delivery of the CST OP model of care, ensuring consistency in the design and implementation of the CST OP model.

## Introduction

The global demographic shift towards an ageing population necessitates a paradigm change in healthcare delivery to meet the complex and diverse needs of older people [1,2]. This demographic change is particularly felt in Ireland, where increases in the ageing population are more significant compared to other EU countries [3]. Older people often experience a combination of physical, cognitive, and psychosocial challenges, making the healthcare requirements of this group intricate and multifaceted [4]. Chronic diseases, functional limitations, and increased risk of adverse health events contribute to the complexity of care for this demographic [5].

A comprehensive and coordinated approach is essential to address these challenges effectively, with the traditional fragmented healthcare model proving inadequate in addressing the multifaceted needs of this growing ageing demographic. Drawing on an integrated care approach, an evidence-based policy response to support optimal outcomes for older people sees a shift away from a functional organisation of care approach, towards delivering care through multidisciplinary teams who are central to navigating this complexity while ensuring efforts are supported to focus on patient-centred and timely access care [6,7,8,9,10,11].

In terms of coordinated community-based efforts from an Irish policy context, Sláintecare has responded to the policy implications of this ageing demographic, focusing on the provision of responsive services to enhance service improvement for older people [12].

Recognising the necessity for an integrated community-based approach, this paper explores the Community Specialist Team for Older People (CST OP) service model for implementing a community-based multidisciplinary approach to provide timely coordinated care to older people with complex care needs living in Ireland.

Community-based multidisciplinary approaches involve the collaboration of healthcare professionals from diverse fields, including Geriatricians, Physiotherapists, Occupational therapists, Social workers, Dietetics, Speech and language therapists, Psychology, Pharmacy and Nursing. Team composition can be varied and is guided by regional and recruitment issues. By consolidating expertise in one location, these multidisciplinary approaches aim to provide integrated care tailored to the specific needs of older people promoting continuity of care, reducing fragmented services, and enhancing the overall patient experience [13]. The concept of delivering timely co-ordinated care within a defined period is key to optimising healthcare outcomes for older people [14].

In Ireland, CST OP represents a core element of the Enhanced Community Care/Integrated Care Programme for Older People (ECC/ICP OP) and National Clinical Programme for Older People (NCP OP). ICP OP aims to reorientate delivery of care away from acute care towards community-based care, emphasising the importance of prompt access to Comprehensive Geriatric Assessment (CGA) and the creation of clear and systematic pathways that encompass cohesive health care services.

The aim of CST OP is to offer timely coordinated care delivered in one place to older people with complex care needs for a defined period of time. There are currently 30 CST OP teams in places across Ireland, with team members typically comprised of a range of health and social care professionals including nurses, physiotherapists, occupational therapists, speech and language therapists, community connectors (connects patients to community resources and opportunities), social workers, and dieticians, with consultant geriatricians playing a key clinical governance role. What is unique to the CST OP is having MDT expertise in one location. CST OP offers an assessment to provide an evaluation of three key areas: medical conditions, functional capacity and social circumstances. The results from this assessment are used to develop a coordinated plan and delivery of care, with the aim of achieving enhanced health outcomes for older people. Drawing on a range of MDT skills, CST OP offers an integrated care MDT approach tailored to the specific needs of older people promoting continuity of care, reducing fragmented services, and enhancing the overall patient experience [13]. International evidence suggests that community-based models of integrated care vary with respect identification of older adults at-risk of poor health outcomes, team composition, setting, intervention components and outcomes reported [15]. Furthermore, components of care integration at the micro, meso and macro levels are inconsistent and largely focus on microclinical care integration processes [15,16]. Thus, further research exploring models of community-based integrated care approaches is warranted [15,17,18].

Similarly, with the growing establishment of these teams and the organic way in which these teams have developed, variation has developed across CST OP sites. It is imperative to uphold fidelity to the service model and ensure robust and consistent design and implementation throughout the country to reduce service variation and support an effective model of implementation.

While the community-based multidisciplinary approaches of CST OP offer benefits, with recent figures reporting almost 100,000 engagements with older people through CST OP teams, which saw 74% of CST OP patients discharged home with community-based interventions as opposed to requiring hospital admission [14].

However, the CST OP service model, given its roll out beginning in 2016, would benefit from exploring and developing a standardised approach to how this service model operates. The rationale for such standardisation emanates from challenges surrounding efficient resource allocation, as well as the need to explore culture change in supporting effective delivery of CST OP in Ireland [19]. Recent research into the CST OP approach echoed this research gap, highlighting the need for greater efforts to support the MDT interprofessional collaboration approach underpinning this service [20,21]. Furthermore, the lack of homogeneity across community-based models of integrated care internationally has implications for the optimum operating model and stakeholder experience.

Through exploring these service model aspects, this paper aims to develop consensus on the core elements of the CST OP service model as a means of addressing and overcoming service variation issues.

## Methods

The process of establishing consensus on the core elements of the CST OP service model utilised a modified Delphi approach, which has previously been effectively applied to provide a systematic way of generating consensus in a range of health service models [22,23,24]. Central to the overall approach was engagement and collaboration with Enhanced Community Care (ECC)/Integrated Care Programme for Older People (ICP OP) and National Clinical Programme for Older People (NCP OP) who served as an advisory group, guiding the development and progress of the study. The NCP OP advisory group was comprised of key stakeholders across national level NCPOP roles, with insights and experiences from CST OP stakeholders to ensure the research aims and approach aligned with service implementation of the model nationally.

This study received ethics approval from the University of Limerick Faculty of Education and Health Sciences Research Ethics Committee (2023_03_10_ER). Written informed consent was obtained from all participants involved.

### World Café Focus Groups

The World Café draws on a Focus Group approach to facilitate the exploration and discussion of topics within diverse groups in a balanced and open environment [25]. World Cafés as a means of qualitative data collection have proven useful in bringing together a range of diverse participants to share and learn through evolving rounds of group discussion [26].

For the purpose of this study, the aim of the World Café Focus Groups was to facilitate discussions across diverse groups of CST OP stakeholders as the first phase of consensus development of core components of the CST OP service model. The World Café Focus Groups provided equal opportunities for participants across a range of disciplines and backgrounds to encourage responses providing a greater understanding of the perceptions of participants on key service elements [27]. The CST OP World Café Focus Group guide was developed following a review of the literature and consultation with the NCP OP advisory group.

Following the development of the World Café Focus Groups guide, a pilot was conducted with CST OP members, resulting in minor revisions made regarding clarification of terminology and phrasing. In addition to the piloting of the World Café Focus Group Guide, a Facilitator Familiarisation Session was held to ensure all facilitators fully understood the process and structure of the World Café approach, ensuring facilitators followed the same process in terms of facilitation, note taking and logistical elements ahead of the World Café. Sixteen facilitators (CF, AH, EA, PH, BC, CH, JH, MM, CD, ÍOS, CD, LB, DL, DH, DM, and RG) were involved in facilitating the World Café Focus Groups, with the support of a scribe for each group. All facilitators followed the World Café Focus Group Guidance and World Café Focus Group Guide to ensure a coherent and systematic approach.

Recruitment of participants for the World Café Focus Groups was informed in the first instance by purposive sampling, with inclusion and exclusion criteria developed and defined in collaboration with ICP OP NCP OP. This focused on current members of CST OP teams or key stakeholders involved in the management or implementation of CST OP teams. Each participant received a Delegate Pack with information on an Overview of the World Café and consensus building process, Worksheet, Study Information Leaflet and Consent Form.

ECC/ICP OP & NCP OP acted as a gatekeeper in the recruitment of participants with all CST OP members and key stakeholders assigned to a mailing list, whereby each CST OP team was represented by three members /stakeholders, receiving a World Café invitation and Delegation Pack. The Pack included a Study Information Sheet and Consent Form which participants could complete and return ahead of the event, with the option for participants to submit Consent Forms on the day of the event, while also ensuring participants had the opportunity to contact a member of the research team ahead of the event with any queries.

In order to capture greater representation from CST OP members who were unable to attend the World Café in person, the Delegate Pack included a Worksheet to ensure all team members had an opportunity to contribute. The Worksheets were shared ahead of the World Café to give CST OP members an opportunity to discuss the core elements of the CST OP service model and facilitate team discussions ahead of the in-person event. In addition to the Worksheets, participants also had the opportunity to take part in a Pre- and Post-Event Survey hosted on Qualtrics, as another means of ensuring CST OP members who could not attend in person were provided with the opportunity to shape the World Café discussion. This information was included in the qualitative data analysis along with the data collected at the World Café Focus Groups. Information from the Worksheet and the Pre- and Post-Event Survey were used to capture insights from broader CST OP teams in shaping the development of consensus on CST OP nationally. A total of 97 CST OP key stakeholders took part in the World Café Focus Groups.

### Real-Time Delphi Survey

Following the World Café Focus Groups (n = 7 groups, n = 97 participants), data from the Worksheets, Pre-Event Survey, Facilitator, and scribe notes were analysed (CF, RG) utilising a reflexive thematic approach [28] to produce content for phase two of the consensus building process of a Delphi questionnaire.

Qualitative data analysis was conducted (CF), NVivo 12 was used to support the management and analysis of data. An inductive approach of coding as outlined by Braun and Clarke was followed to develop themes [29]. Members of the research team (CF and RG) worked in compliance with guidance to ensure consistency in coding process as well as the production of preliminary themes.

Four key themes were produced utilising qualitative data from the World Café Focus Groups: Fundamentals of service design (Aims, Objectives, Outcomes, Target patient population, Referral Pathways), Access and transitions of care (Single point of access, Integration and capacity, Onward referral, Common Assessment), Team Functions (Internal Clinical Governance, Operational Governance, Internal Team Processes) and Quality improvement (Service evaluation).

The Delphi method seeks to integrate the benefits of group participation, including diverse viewpoints and expertise, while avoiding the limitations imposed by face-to-face social interactions [30]. A Real-Time Delphi (RTD) approach was selected for this study as it best aligned with the multi-levels of engagement and resource awareness in the development of consensus for the CST OP service model. First developed by Gordon and Pearse, a RTD approach was developed to improve the efficiency of the Delphi process, with a particular focus on minimising time and resource constraints [31]. The RTD approach captures participants responses in real time, allowing participants to view overall responses and providing the opportunity to revise responses in order to reach consensus [32].

Design of the Delphi survey was informed by the four key themes produced from World Café Focus Groups qualitative data. Through consultation with NCP OP, a series of statements were developed, with the Qualtrics platform utilised to administer the survey (Additional file 1). Participants had the opportunity to rank 74 statements using a 9-point Likert scale. Participation in the Delphi survey was only open to CST OP key stakeholders who had previously been involved in the World Café Focus Groups (n = 97). The CST OP RTD remained open for six weeks, with reminder emails sent to participants fortnightly.

### Analysis

RTD analysis was conducted using JASP statistical software. Responses from the RTD were subjected to descriptive and frequency statistical calculation for each ranking entry (AW). Analysis drew upon the 9-point Likert scale entry. Each RTD statement was examined to include consensus criteria comprising of (1) ≥70% of the entry ranked 7–9, (2) mean entry rank 7–9, and (3) median entry rank 7–9 with an inter-quartile range <3. The overall consensus criteria were identified by utilising the most exact of three criteria as outlined (median 7–9, IRQ <3).

## Results

**Figure 1:**
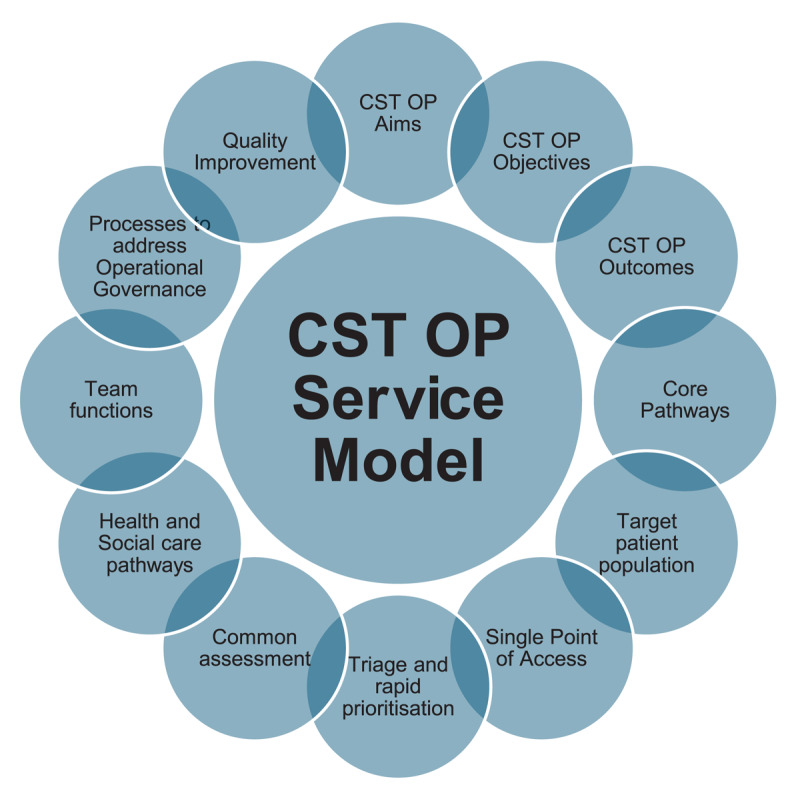
CST OP Service Model Consensus.

A total of 77 participants completed the CST OP RTD survey. Based on the initial 97 participants who were invited to take part in the survey, this represented a response rate of 78%. Participant profile data captured from the 77 RTD survey participants captured representation across key groups including, physiotherapy, geriatrics, dietetics, nursing, occupational therapy, speech and language therapy, social work, and GP. On completion of CST OP RTD survey response analyses, the NCP OP advisory group and the research team convened to review the final list of consensus statements (Figure 1). Consensus criteria was used to inform this process, with the group applying the strictest consensus criteria (median 7–9, IQR) in this evidence-based decision-making process. Additional information on the complete final list of CST OP consensus statements are outlined in Additional file 1.

### Aims of CST OP

The statements agreed upon defining the aims of CST OP service model focused on delivering an age attuned comprehensive multidisciplinary service which maximises independence and enables older people to live well at home while also providing timely access to a specialist multidisciplinary assessment in the community (CGA) to identify and manage the drivers of frailty.

### Objectives CST OP

In terms of consensus regarding the objectives of CST OP, this comprised of supporting frail older people to live well at home and in their communities through providing a timely response; aiming to have first contact initiated within 24 hours from referral.

### Outcomes

CST OP outcomes included recognition of CST OP evaluation, including a measure of patient experience, with evaluation of CST OP recognised as a core element of enhancing patient experience. The need for standardisation of CST OP key performance indicators was also deemed to be required nationally.

### Core pathways

Consensus on statements regarding core pathways included the need for further integration of CST OP services with supports to live well, as well as further integration with chronic disease pathways.

### Target patient population

Target patient population consensus statements included the need for CST OP to incorporate target older people with comorbidities indicative of frailty, older people >65 years living with or at risk of frailty. CST OP was seen as to give equal priority to referrals from GPs and acute services.

### Single point of access

Consensus regarding single point of access comprised of CST OP providing a single access point for referrals. In addition to referral screening, triage time was seen as essential to support timely service access.

### Triage and rapid prioritisation

Triage and rapid prioritisation consensus included explicit criteria on referral source, age and diagnostic criteria is required to support an efficient triaging process. Agreement also included triage which can be carried out by any clinical CST OP team member with the necessary skill set if eligibility criteria are agreed by team.

### Common assessment

CST OP should use an interdisciplinary assessment proforma as the basis to inform the intervention plan within the team. Consensus statements for common assessment comprised of CST OP adopting a comprehensive geriatric assessment (CGA) and care planning to incorporate a biopsychosocial process of assessing frailty, co-morbidity, polypharmacy, cognition, function and mobility, balance, continence, nutrition, psychological and social status.

### Health and Social care pathways

Mapping of local service assets, supports and resources across the Older Persons Service Model facilitates the function of the CST OP and integrated and collaborative working practices. Health and social care transitions of care and pathway mapping should include onward referral and access to other services as communicated and agreed across services. It should also include clear CST OP discharge criteria to support a more streamlined and efficient CST OP service.

### Team functions

Consensus statements for team functions covered the involvement of older people/advocacy groups in CST OP governance as a support for patient centred approach. Appropriate clinical and operational leadership to develop and design services is supported and implemented was also deemed as a core consensus statement.

### Processes to address Operational Governance

Processes to address operational governance statements comprised of the operational policy including a matrix of accountability to outline how dual operational and clinical accountability mechanisms operate. A clear operational policy, developed and agreed by team members and supported by the organisations high-level local governance was identified as critical to outlining how the CST OP team operates.

### Quality Improvement

Consensus statements for CST OP quality improvement (QI) included the role of QI in enhancing patient experience and outcomes while also agreed as important to enhance staff experience. A national competency framework was seen to be required specific to CST OP.

## Discussion

At the core of this paper are a range of insights into the service landscape which capture challenges regarding access, fragmentation of service, coordination, and continuity of care, echoing previous policy guidance surrounding practice priorities [33]. While the CST OP service model offers an effective community-based MDT integrated care approach, a clear understanding of the core components and context of the service model is key to supporting best practice with this model for stakeholders and patients.

### Consensus Overview

The MDT composition of CST OP teams brings both benefits and challenges, given the diverse range of HSCPs, the need for clarification and consistency in core elements of the service such as aims and outcomes to ensure a robust and clear service delivery approach, of which consensus is a key function in facilitating [34,35].

Through the real-time Delphi (RTD) approach, consensus was developed on the core elements of the CST OP service model. The findings of this study result in a range of core consensus statements of the CST OP service model. Utilising a RTD approach, a national panel of CST OP key stakeholders reached consensus on the core elements of the CST OP model of care [36]. The final list of statements reflects key service elements, providing clinicians with a guiding framework to ensure homogeneity with respect to implementation and evaluation of the CST OP service model, to support a more standardised approach to the CST OP service, echoing the challenges standardising integrated care and system efforts in meeting service deliverables [37,38]. These key areas align with World Health Organization (WHO) guidance on the integration of a people-centred health service framework [23].

One of the key areas emerging from the Delphi process was agreement amongst participants on core functions of the CST OP service model; aims, objectives and target population – clarity and consistency regarding these three key areas had been lacking. Given that older adults are clinically complex and heterogeneous, the identification of a population of interest for individualised comprehensive assessment and interventions may be challenging. Findings from a recent prospective cohort study of 303 older adults who underwent a community-based integrated model of care underpinned by the components of CGA by a CST OP team, reported that older adults who screened positive for frailty were at significantly increased risk of adverse outcomes including functional decline, hospitalisation, ED presentation and mortality at six months [39]. Similarly, our consensus-based statements included a target population of older adults who identified as living with frailty.

The area of fundamentals of service design gave insights into the process CST OP referrals as well as aims, objectives, and outcomes of CST OP teams. Insights on experiences surrounding CST OP referrals featured across groups, focusing on referral criteria and referral sources. This need to recognise the context surrounding referral mechanisms and approaches to best match the overall aim of the service has previously been established in the literature [40].

The consensus reflected a shared recognition of the broader challenges faced by CST OP in effectively meeting the needs of the target demographic. CST OP teams were acknowledged as providing valuable work for older people in a service that is newly established, with the team approach and person-centred components recognised reflecting a people-centred integrated care approach [41]. In line with our study, consensus regarding a ‘person-centred’, ‘collaborative’ and ‘preventative’ approach was previously found in two Delphi consensus studies related to integrated care [42,43]. Another Delphi study by Briggs et al. (2018) reported consensus on the necessary actions to implement the WHO’s Integrated Care for Older People (ICOPE) approach [16]. Similar to our findings, this Delphi study reported consensus regarding case management, care plans, geriatric assessments and interdisciplinary team working [16]. In contrast to our findings, this study by Briggs et al. (2018) found consensus on the use of data sharing information systems, something that has previously been reported as an enabler to integrated care at the macro level [44].

Areas that were identified to further strengthen the services included capacity issues, reflecting similar capacity challenges captured in previous research [45]. The need for greater engagement and communication to ensure the patient is not adversely impacted was highlighted. Issues related to communication with patients and between healthcare professionals were previously reported as barriers to successful delivery of community-based CGA, an operational model of integrated care [46]. Furthermore, a lack of clear communication pathways was reported as a key driver to increased fragmentation of care from the acute to the community setting [47]. Accordingly, our consensus-based statements included objectives that older adults’ needs and concerns are communicated effectively.

Variation across team composition was evident across sites. This variation mirrors broader heterogeneity across CGA based service models, with the need to fully explore and outline specifics in terms of core elements of what CGA services such as CST OP offer, how these are delivered and evaluated [48]. Our consensus-based statements focussed on integrated, CGA and care planning, case management, advocacy and interventions tailored to what matters most to older adults on discharge from the acute as well as within the community setting. Given the high rates of adverse outcomes experienced by older adults living with frailty [39,49], evidence-based holistic models of integrated care in the community setting to support hospital avoidance strategies are a key priority for researchers, clinicians and policymakers. A recent systematic review of 22 randomised controlled trials recruiting 7219 community-dwelling older adults examined the effectiveness of home based CGA [18]. This study reported that home-based CGA improves clinical and process outcomes for community dwelling at-risk older adults, but called for further research to explore community-based models of CGA given the high levels of heterogeneity across interventions delivered [18]. The CST OP model of care represents an integrated and comprehensive approach to healthcare delivery in the community setting; uniformity in implementation of this model of care will facilitate robust reporting and evaluation of its efficacy and effectiveness.

The majority of statements that did not achieve consensus were related to health and social care pathways. Most notably, the survey revealed that community and outpatient services to support older adults to live will following discharge from CST OP services were inadequate. This mirrors the international evidence base whereby a lack of efficient access to services, a comprehensive overview of local health and well-being facilities was seen as a barrier to the successful implementation of community-based CGA [50]. This may be further explained by the demand for services exceeding the capacity of the MDT to deliver care [51].

This study supports findings on the Irish health system reform which highlighted a need to adopting a standardised team-based service model approach to effectively implement integrated health and social care services [7]. Our findings support the need for a clear and robust process to support interdisciplinary approaches to prevent and manage frailty in the community. Considering the various typologies of integrated care for older adults and the recent shift of healthcare delivery towards an integrated approach in the community setting internationally, this consensus marks a significant advancement in establishing national standards for the optimum operating model.

### Future research opportunities

The consensus statements developed for the CST OP service in Ireland are viewed as a starting point in a long-term approach of collaboration and engagement with key stakeholders in this area. It is envisaged that the consensus statements developed as result of the World Café Focus Groups and RTD survey will be revisited at agreed time points to reflect and capture changes within the service as well as the broader national health landscapes. Variations in outcome reporting across models of integrated care has impacted the synthesis of findings in recent literature reviews [17,18]. Thus, impacting the generation of assertions regarding efficacy and policy recommendations. In recognition of the need to a more streamlined and systematic approach to broader CGA based health care models, utilising a Core Outcome Set (COS) approach would build upon findings from this study, incorporating the core elements captured to encompass outcomes that should be measured and reported in all CGA based models in clinical trials.

### Limitations

A limitation of this study was the lack of opportunity for the lived experience to be incorporated. The engagement of CST OP service users and carers in the consensus development process was identified as a key area for capturing patient perspectives in addition to policy and practice insights [23]. To address this limitation while incorporating key learnings from a qualitative study of older people and family carers who have availed of the CST OP service [47], the CST OP consensus findings are being used to develop a service user experiential survey. This survey has been piloted and will be used in an ongoing study to capture the perspective of the CST OP service from a patient and carer perspective. The key domains from the consensus development process have been utilised in this survey, findings of which will further contextualise the consensus findings and inform the implementation of the CST OP service model.

### Implications for practice

The findings of this study contribute valuable insights to the discourse on supporting older people age in place. By capturing the perspectives from participants from a range of Health and Social Care backgrounds, this study provides a robust foundation for future research and policy development in the field of older adult healthcare. Moving forward, efforts should be directed towards translating these consensus-driven recommendations into actionable strategies, fostering collaboration among stakeholders, and addressing the identified challenges to facilitate the widespread implementation of CST OP. The creation of a consensus-driven CST OP service model is a core element towards establishing national standards for CST OP to support the development, implementation, and evaluation of the CST OP model in a standardised and transparent approach to minimise service variation and maximise patient outcomes.

## Conclusion

The RTD process has resulted in the refinement and agreement of a set of core CST OP statements that outline the key components of the CST OP service model. These key CST OP statements will be used to guide future implementation and delivery of the CST OP model of care, ensuring consistency in the design and implementation of the CST OP model, while also supporting a robust and consistent approach within the older person service model across an integrated care pathway.

## Additional File

The additional file for this article can be found as follows:

10.5334/ijic.8781.s1 Additional File 1. Final list of CST OP consensus statements.
