# Supplementary material for: Community Specialist Teams for Older People Consensus Development: A Real-Time Delphi Approach
Source: Int J Integr Care. 2025 Oct 21;25(4):3. doi: 10.5334/ijic.8781 (PMC12551642; doi:10.5334/ijic.8781)
Supplement: Additional File 1. — Final list of CST OP consensus statements. [file ijic-25-4-8781-s1.pdf]

Additional file 1: Final list of CST OP consensus statements

Consensus Criteria				
	**% scoring 7-9 (≥70)**	**Median 7-9, (IQR < 3)**	**Mean 7-9**	**Rank (Based on Mean)**
**Aims of CST OP Teams**				
To provide timely access to a specialist multidisciplinary assessment in the community (CGA) to identify and manage the drivers of frailty	90.91	9	8.242	2
To provide a specialist episode of care focusing on preventing and resolving a crisis for older people at risk of frailty	87.88	9	8.030	4
To deliver an age attuned comprehensive multidisciplinary service which maximises independence and enables older people to live well at home	90.92	9	8.348	1
To work in partnership with older people/families to coordinate and provide enhanced health and social care services in their home and communities	86.37	9	8.121	3
**Objectives CST OP Teams**				
To provide an age attuned egress pathway facilitating early supported discharge from the acute services	59.68	7	6.516	6
To support frail older people to live well at home and in their communities	92.07	9	8.397	1
To provide a timely response aiming to have first contact initiated within 24 hours from referral	42.63	6	5.984	
To promote positive healthy ageing through comprehensive geriatric assessment (CGA) case management and tailored interventions	88.89	9	8.206	2
To implement a person centred holistic integrated care planning approach to health and social care services e.g. CHNS and voluntary agencies	79.04	9	7.871	4
To actively support patient advocacy ensuring frail older adults' needs and concerns are addressed and clearly communicated with facilitated input from family / carer	82.54	9	7.871	4

To actively support patient advocacy ensuring frail older adults' needs and concerns are addressed and clearly communicated	85.49	9	8.032	3
To facilitate supported patient discharge from CST OP to local support to live well services	78.69	9	7.607	5
**Outcomes**				
Evaluation of CST is important to enhance patient experience	91.67	9	8.250	2
Evaluation of CST is important to enhance staff experience	81.67	9	7.850	4
Greater resources and training are required to fully enhance understanding and value of CST OP evaluation by team members	86.43	8	7.814	5
Evaluation of CST OP should include process outcomes only e.g., incidence of ED (re)presentation, conversion to hospital admission	38.19	5	4.818	9
Evaluation of CST OP should include both process and clinical outcomes e.g., incidence of 30-day unscheduled ED revisit and an ADL index measure	72.88	9	7.508	6
Evaluation of CST OP should include a measure of patient experience of CST OP	96.61	9	8.525	1
Standardisation of CST OP key performance indicators is required nationally	91.67	9	8.233	3
The CST OP should report quarterly metrics to the HSE National EC	59.65	8	6.632	7
The CST OP should report biannual metrics to the HSE National ECC	42.31	5	5.788	8
**Core pathways**				
Further integration of CST OP services with primary care is warranted	87.73	9	8.088	3
Further integration of CST OP services with chronic disease pathways is warranted	91.22	9	8.105	2
Further integration of CST OP services with FFD is warranted	78.57	9	7.929	4
Further integration of CST OP services with acute services is warranted	76.79	9	7.661	5
Further integration of CST OP services with supports to live well is warranted	87.72	9	8.246	1
**Target patient population**				

CST OP should target older adults >65 years living with or at risk of frailty	69.64	8	7.250	2
CST OP should target older adults >75 years living with or at risk of frailty	62.26	8	6.377	4
CST OP should target older adults with comorbidities indicative of frailty	72.73	8	7.364	1
CST OP should prioritise referrals from general practitioners	48.15	6	5.704	5
CST OP should prioritise referrals from acute services	34.18	5	5.185	7
CST OP should give equal priority to referrals from GPs and acute services	60.38	8	6.679	3
CST OP should accept self-referrals to the service	17.64	2	3.196	8
CST OP should accept referrals from any HCP in the acute or community setting	49.06	6	5.509	6
**Single point of access**				
A Single Point of Access (SPA) which would consist of one referral route for patients requiring CST OP (e.g. Healthlink) is essential to support a standardised CST OP service model	82.46	8	7.719	3
A SPA should only be managed by clinicians to screen referrals	61.82	8	6.655	4
A standardised approach to CST OP referral screening and triage time is essential to support timely service access	82.46	8	7.860	2
The CST OP should provide a single access point for referrals	87.5	9	8.250	1
**Triage and rapid prioritisation**				
Triage can be carried out by any clinical CST OP team member with the necessary skill set if eligibility criteria are agreed by team	87.28	9	8.291	2
The necessary information skill set required to triage is based on the clinical, health & social care information provided (frailty score, team judgement, risk rating)	89.28	9	8.071	4
There should be a timeline for response based on efficient prioritisation by the person(s) who triages	91.07	9	8.161	3
Explicit criteria on referral source, age and diagnostic criteria is required to support an efficient triaging process	96.43	9	8.643	1

**Common assessment**				
CST OP should conduct a comprehensive geriatric assessment (CGA) to incorporate a biopsychosocial process of assessing frailty, co-morbidity, polypharmacy, cognition, function and mobility, balance, continence, nutrition, psychological and social status	94.55	9	8.600	1
CST OP should use an interdisciplinary assessment proforma as the basis to inform the intervention plan within the team	84.62	9	7.942	2
A standardised CGA should be issued nationally across all CST OP sites	79.24	9	7.811	3
**Health and Social care pathways**				
Integration of care is hampered by the issue of access to diagnostics in the community	83.02	8	7.321	5
There is a need for standardised criteria for outward referrals and discharge protocols to support a more streamlined and efficient CST OP service	79.62	8	7.537	3
There is adequate community and outpatient services to support older adults following discharge from CST OP services	6.12	3	3.347	
Care pathways should include onward referral and discharge criteria in the older persons transition of care to support a more streamlined and efficient CST OP service	83.03	8	7.811	2
National guidance on methods to complete local asset mapping is warranted	77.77	8	7.370	4
Mapping of service assets locally supports the function of CST OP	90.56	8	8.000	1
An up to date assets map of local older peoples services and supports to live well should be the responsibility of each CST OP site	43.40	6	5.585	7
An up to date assets map of local older peoples services and supports to live well should be the responsibility of national CST OP structures	48.15	6	6.167	6
**Team functions**				

The involvement of older adults/advocacy groups in CST OP Governance supports a patient centred approach	88.68	9	8.321	1
Shared population clinical data for planning and a collaborative approach to measuring performance	80.39	8	7.255	3
Appropriate clinical and operational leadership to develop and design services is supported and implemented	88.69	9	8.132	2
**Processes to address Operational Governance**				
Establishing a high-level local governance structure across health and social care, community, acute and the third sector with senior sponsorship	92	8	7.920	7
The Governance Group is a high-level group that is there to guide a local implementation group LIG, enable decision making locally, with a mechanism to escalate issues requiring national input when required	92	8	8.000	6
A Memorandum of Understand (MOU) developed and agreed, formalising relationships and multi-level partnerships	87.75	9	8.163	3
A clear operational policy, developed and agreed by team members and supported by the organisations high-level local governance is critical to outlining how the team operates	92.31	9	8.250	2
The operational policy will include a matrix of accountability to outline how dual operational and clinical accountability mechanisms operate	92.17	9	8.275	1
A collaborative co-designed joint forum with all key stakeholder involved, including patient/carer input	88	9	8.020	5
A National communique detailing the roles and responsibilities of each respective service is needed	84.31	9	8.078	4
**Quality Improvement**				
There is a need for an UpToDate feedback system for teams reporting to local governance structures	80	8	7.400	6
The need and value of QI is well understood by team members	68	7	7.020	8
QI of CST OP is important to enhance	98.05	9	8.431	1

patient experience and outcomes				
QI of CST OP is important to enhance staff experience	94	9	8.300	2
Greater resources and training are required to fully enhance understanding and value of QI of CST OP by team members	80	8	7.560	5
Research is a key component of the CST OP model	84	8	7.660	4
Research is seen as a priority by CST OP team	46.95	6	5.510	11
Research is valued in the CST OP model	62	7	6.640	10
Greater efforts are required to enhance understanding and value of research	72	7	6.840	9
The research agenda needs to be framed by NCP OP Research Strategy	81.64	7	7.306	7
A national competency framework is required specific to CST OP	82	8.5	7.900	3
